# Polarization in the COVID‐19 pandemic: The impact of vaccination status and conspiracy theories

**DOI:** 10.1111/nyas.15408

**Published:** 2025-08-04

**Authors:** Lisa Willemsen, Chiara Serafini, John Butler, Sümeyye Ergün, Jan‐Willem van Prooijen

**Affiliations:** ^1^ Department of Social Psychology University of Groningen Groningen The Netherlands; ^2^ Vrije Universiteit Amsterdam Amsterdam The Netherlands; ^3^ NSCR Amsterdam The Netherlands; ^4^ Maastricht University Maastricht The Netherlands

**Keywords:** conspiracy beliefs, conspiracy theories, COVID‐19, dogmatic intolerance, hate, polarization, vaccination

## Abstract

The COVID‐19 pandemic gave rise to a new social identity through vaccination status and yielded substantial polarization between vaccinated and unvaccinated people. Across two studies conducted via online platform Prolific, we examined the polarization between vaccinated and unvaccinated people in the United States. Study 1 (*N* = 646) revealed that vaccinated people were more polarized toward unvaccinated people than vice versa across a range of polarization indicators. Conspiracy beliefs moderated these effects. Higher conspiracy beliefs predicted increased polarization among unvaccinated people; however, lower conspiracy beliefs predicted increased polarization among vaccinated people. Study 2 (*N* = 503) focused specifically on feelings of hate and revealed that vaccinated and unvaccinated people were polarized toward different target groups. While vaccinated people expressed more hate toward unvaccinated people than vice versa, unvaccinated people expressed more hate toward the government. Additional analyses indicated that besides public health concerns, feelings of hate also predicted vaccinated people's support for excluding unvaccinated people from public places. These findings offer novel insight into the complex dynamics that help explain the polarization that occurred among both vaccinated and unvaccinated people.

## Graphical abstract

We examined polarization of vaccinated versus unvaccinated people during the COVID‐19 pandemic. Study 1 suggested that vaccinated people were more polarized toward unvaccinated people on a range of indicators. Higher conspiracy beliefs predicted increased polarization among unvaccinated people, but lower conspiracy beliefs predicted increased polarization among vaccinated people. Study 2 suggested that while vaccinated people were polarized more toward the unvaccinated, unvaccinated people were polarized more toward the government.

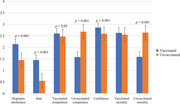

## INTRODUCTION

The COVID‐19 pandemic drastically altered societies around the world. Billions of people were forced to stay at home to minimize social contact. Wearing face masks and physical distancing became the new norm.^1^, ^2^ Besides imposing restrictions to protect public health, however, the pandemic also stimulated substantial polarization among people, which was reflected in protests and public hostilities.^3^, ^4^ A key factor that appeared to have polarized groups of people was deciding whether to get vaccinated with one of the mRNA vaccines.^5^, ^6^ In this way, the COVID‐19 pandemic gave rise to a new social identity through vaccination status.^7^, ^8^ People carried vaccination certificates with them to access public spaces, and dating apps allowed users to input their vaccination status on their profile. Previously, vaccination status (e.g., childhood vaccines) had been an individual and private identity. Now it became a publicly shared group identity.^7^

This new public identity had significant implications for how vaccinated and unvaccinated people perceived each other. Choosing to be vaccinated or not had real‐world implications.^7^, ^8^ Multiple countries restricted access to public spaces for unvaccinated people (e.g., having to show proof of a recent negative diagnostic test, a vaccine certificate, or a recovery certificate). This may have resulted in feelings of oppression and disadvantage among unvaccinated people, whose movements were most restricted. Vaccinated people, in turn, were likely to blame unvaccinated people for the continued lockdowns (i.e., curfews, face masks, social distancing). Both vaccinated and unvaccinated people hence had reason to feel threatened by the other group, and such perceived intergroup threat can increase polarization.^9^

The current research sought to examine polarization between vaccinated and unvaccinated people during the COVID‐19 pandemic. Prior research found that vaccination status indeed predicted polarization between vaccinated and unvaccinated people.^7^, ^8^ Vaccinated people, in particular, aimed to disadvantage unvaccinated targets in a resource allocation task.^8^ We aimed to expand on this research and explore what polarization among vaccinated and unvaccinated people looked like by examining a variety of affective and cognitive polarization indicators. Additionally, we examined the moderating role of conspiracy theories in the polarization between vaccinated and unvaccinated people. Past studies have found that conspiracy beliefs are associated with vaccine hesitancy^10^, ^11^, ^12^ and decreased inoculation,^13^ but also impact how vaccinated and unvaccinated people polarize toward each other.^6^

### Vaccination and intergroup polarization

Broadly defined, intergroup polarization is the process in which a social group divides into two subgroups with conflicting positions, goals, and viewpoints.^14^ Such polarization is rooted in group identity^15^ and affect.^16^ Group membership drives human behavior^8^, ^17^, ^18^ and is aimed at reaffirming ingroup superiority.^7^ The more strongly a person identifies with their ingroup, the more they dislike and distrust dissimilar outgroups. In this way, high group identification can predict increased polarization toward outgroup members.^9^, ^16^, ^19^, ^20^ We identified two key dimensions of intergroup polarization—affective and cognitive polarization—and examined how vaccinated and unvaccinated people differed across these dimensions.

Choosing not to be vaccinated has been associated with moral outrage among vaccinated people.^21^ As such, vaccinated people might display greater affective polarization, defined as the extent to which people hold negative feelings about the outgroup. We have operationalized affective polarization as dogmatic intolerance (the tendency to reject, and consider as inferior, any ideological belief that differs from one's own)^22^, ^23^ and feelings of hate.^24^ Unvaccinated people may mostly view the institutions that administer the vaccine and the government that imposes restrictions—rather than vaccinated people themselves—as conflicting with their goals. Hence, we expected them to display less affective polarization toward vaccinated others.

Cognitive polarization refers to the hostile appraisals and cognitions that people may hold about a different group, notably intergroup stereotypes, and superiority appraisals in the form of overconfidence. Morality and competence perceptions are important dimensions of intergroup stereotypes.^25^ As to morality, research shows that vaccination is perceived as a social contract^26^, ^27^ and a collective action intention^7^ with the goal of keeping others safe. Unvaccinated people may be perceived as choosing to put others around them at greater risk, thereby breaking the social contract. Indeed, there is evidence suggesting that vaccinated people attributed more responsibility for infection when the infected person was unvaccinated, viewing them as less moral and trustworthy.^28^ Among vaccinated people, there was a link between viewing vaccination as a prosocial act and perceiving unvaccinated people as threatening to public health.^8^, ^26^ This suggests that vaccinated people may perceive unvaccinated people as less moral than vice versa.

As to competence, unvaccinated people believed the COVID‐19 vaccines to be threatening to public health.^29^, ^30^, ^31^ Vaccine hesitancy has been linked to mistrust in institutions and science.^32^, ^33^, ^34^ As such, unvaccinated people might consider vaccinated people gullible for accepting the health advice supported by science, which they may see as a form of propaganda.^35^ We therefore expected that unvaccinated people may perceive vaccinated people as less competent than vice versa.

By the same token, we anticipated that unvaccinated people would be more overconfident in their knowledge of the COVID‐19 vaccine. Overconfidence occurs when a person's subjective confidence is higher than their objective performance and abilities.^36^, ^37^, ^38^ While both vaccinated and unvaccinated people may be confident in their knowledge about the COVID‐19 vaccine, the knowledge of unvaccinated people is likely to be rooted in misinformation and conspiracy theories. Such conspiracy beliefs are associated with overconfidence in the form of the illusion of explanatory depth.^39^ Put differently, we expected that particularly unvaccinated people would be overconfident about their knowledge of the mRNA vaccines.

Based on the above arguments, we hypothesized that vaccinated people would show higher levels of dogmatic intolerance (Hypothesis 1a) and hate (Hypothesis 1b) toward unvaccinated people than vice versa. We also expected that vaccinated people would perceive unvaccinated people as less moral, whereas unvaccinated people would perceive vaccinated people as less competent, than vice versa (Hypothesis 1c). Finally, we predicted that unvaccinated people would be more overconfident in their COVID‐19 vaccine knowledge compared to vaccinated people (Hypothesis 1d).

### Conspiracy beliefs and polarization

The pandemic inspired many conspiracy theories—that the virus was a bioweapon created by the Chinese government, or that Bill Gates put microchips in the vaccines to be able to track people. Such conspiracy theories about COVID‐19 had a significant impact on society.^2^, ^30^, ^40^ Conspiracy theories are defined as explanatory beliefs rooted in the assumption that a group of actors have colluded in secret to attain nefarious goals.^2^, ^13^, ^33^, ^40^, ^41^, ^42^, ^43^, ^44^, ^45^ The relationship between conspiracy beliefs and vaccine hesitancy has often been interpreted as evidence that conspiracy theories increase hesitancy. Yet, recent findings suggest that the link to some extent emerges because people use conspiracy beliefs to justify their existing vaccine hesitancy.^12^

Conspiracy beliefs are associated with hostile intergroup perceptions (e.g., stereotypes),^46^ perceived outgroup threat,^47^ polarization,^48^, ^49^ and radicalization.^44^ Research also found that feelings of coercion (e.g., through government policies stimulating vaccination) promoted increased conspiracy beliefs among unvaccinated people.^6^ Spreading conspiracy theories is associated with fear for stigmatization and exclusion^50^ and research has shown that this fear is justified. COVID‐19 conspiracy beliefs predicted more exclusion experiences 8 months later.^51^

Such exclusionary experiences also characterized the relationship between vaccinated and unvaccinated people. Studies have shown that vaccinated people are more likely to discriminate against unvaccinated people^5^, ^8^ and polarize against them^6^ rather than vice versa. There is ample evidence suggesting that disbelieving conspiracy theories is associated with rejecting conspiracy theorists.^50^, ^51^ Our expectation was that COVID‐19 conspiracy beliefs would predict polarization among both vaccinated and unvaccinated people, but in different ways. We expected that higher conspiracy beliefs would predict higher levels of polarization in unvaccinated people.^6^ Consistent with the notion that rejecting conspiracy theories is associated with rejecting conspiracy theorists,^50^, ^51^ however, we also expected that lower conspiracy beliefs would predict higher levels of polarization among vaccinated people.

We thus examined conspiracy beliefs as a moderator of polarization in both vaccinated and unvaccinated people. Following our line of reasoning, we expected that conspiracy beliefs would be associated with higher levels of affective and cognitive polarization among unvaccinated people (Hypothesis 2). Among vaccinated people, however, we expected that lower conspiracy beliefs would be associated with higher levels of affective and cognitive polarization (Hypothesis 3).^b^

## HYPOTHESES TESTING: STUDIES 1 AND 2

Study 1 tested Hypotheses 1–3, and found that vaccinated people were polarized more toward the unvaccinated than vice versa. Study 2 then extended on these findings by examining the possibility that unvaccinated people may have been polarized as well, but toward a different target. Specifically, as unvaccinated people may hold the government (and not vaccinated people) responsible for their problems, which include feeling pressured to get vaccinated and being restricted in their access to public spaces, unvaccinated people may have displayed affective polarization (e.g., feelings of hate) mostly toward the government.

Ethical approval was obtained from Vrije Universiteit Amsterdam (VCWE‐S‐21‐00228). Both Study 1 and Study 2 were preregistered on the Open Science Framework prior to data collection. The raw data from both studies, including survey materials and coding scripts, can be found on OSF (https://osf.io/5n4g2/).

### Study 1: Materials and methods

#### Sample

Recruitment took place via Prolific in March 2022. The sample consisted of 646 participants from the United States (35.29% men, 62.38% women, 1.7% nonbinary/third gender) between 18 and 79 years old (*M*_age_ = 38.54, *SD*_age_ = 13.86). A sensitivity power analysis for the effect of vaccination status on polarization (Hypothesis 1) indicates that this sample allows us to detect a small‐to‐medium effect size (*f* = 0.18) with 80% power (*α* = 0.05). We used the Prolific prescreening function to target vaccinated and unvaccinated participants, and slightly oversampled the unvaccinated group to potentially compensate for participants who may have changed their vaccination status since registering on Prolific (*N*_vaccinated_ = 309, *N*_unvaccinated_ = 337). The study took about 8 min and participants were paid 1.00 UKP (approximately 1.28 USD).

#### Procedure

After soliciting participants’ informed consent, we assessed affective polarization (dogmatic intolerance, hate) and cognitive polarization (morality and competence perceptions, confidence in knowledge). Dogmatic intolerance (six items)^23^ and hate (12 items)^52^ were measured on 5‐point scales (0 = *strongly disagree*, 4 = *strongly agree*). The dogmatic intolerance items were tailored toward beliefs about the COVID‐19 vaccine, example item: “I believe everyone should think like me about the COVID‐19 vaccines” (*α* = 0.79), see Supporting Information for a measurement invariance test showing that the scale has configural, metric, and scalar invariance. The hate scale measured anger, contempt, and disgust toward people with a different vaccination status, example item: “I cannot control my anger toward vaccinated (unvaccinated) people” (*α*_vaccinated_ = 0.93, *α*_unvaccinated_ = 0.85).

We then measured morality and competence perceptions for vaccinated and unvaccinated people on 5‐point scales (0 = *extremely low*, 4 = *extremely high*; 11 items).^53^ Five items were used to assess competence perceptions, example item: “How competent are people who choose to get vaccinated [not get vaccinated]?” (*α*_competence vaccinated people_ = 0.96; *α*_competence unvaccinated people_ = 0.96). As we measured confidence in knowledge as a separate variable, we excluded the perceived confidence item. Six items were used to assess morality perceptions, example item: “How friendly are people who choose to get vaccinated [not get vaccinated]?” (*α*_morality vaccinated people_ = 0.93; *α*_morality unvaccinated people_ = 0.95).

Confidence in knowledge was measured by assessing factual knowledge questions about COVID‐19 vaccines and then assessing participants’ confidence in their knowledge. Participants responded to 10 factual knowledge questions in True/False format (KR‐20 = 0.66), see Supporting Information for item‐level difficulty. We calculated the number of correct answers as an indicator of factual knowledge, example item: “Getting vaccinated prevents you from contracting COVID‐19.” After each knowledge question participants rated how confident they felt about their answer on a 5‐point scale (0 = *not confident at all*, 4 = *extremely confident*; *α* = 0.80).

We then assessed conspiracy beliefs. Conspiracy beliefs about the COVID‐19 vaccines were measured on a 6‐item scale, example item: “Pharmaceutical companies are dishonest about the possible dangers of the COVID‐19 vaccines.” Participants rated their agreement on a 5‐point scale (0 = *strongly disagree*, 4 = *strongly agree*; *α* = 0.97). For completed scale items, see the Supporting Information. Finally, we asked participants for basic demographic information.

### Study 1: Results

As preregistered, we conducted a series of ANOVAs to test the effect of vaccination status on polarization (Hypotheses 1a to 1d), and linear regressions with simple slope analyses to test the effect of conspiracy beliefs on polarization across the vaccination status groups (Hypotheses 2 and 3). We centered the continuous independent variables and effect‐coded vaccination status (unvaccinated = −1, vaccinated = 1).

While the primary analyses reported below were without covariates (which was in line with our preregistration), an ANOVA found that participants in the vaccinated group (*M* = 2.37, *SD* = 2.23) reported more liberal views, whereas participants in the unvaccinated group (*M* = 4.75, *SD* = 2.31) reported more conservative views, *F*(1, 644) = 176.50, *p* < 0.001, $\eta_{p}^{2}$ = 0.22. We therefore ran the primary analyses with political orientation, gender, and age as covariates to test the robustness of our results and reduce omitted‐variable bias. These results can be found in the Supporting Information. All hypotheses were still supported after including these covariates. Only some of the unpredicted main effects changed.

#### Effect of vaccination status on polarization

##### Affective polarization

Participants in the vaccinated group (*M* = 2.14, *SD* = 0.78) were more dogmatically intolerant about people with different views about COVID‐19 vaccines than participants in the unvaccinated group (*M* = 1.45, *SD* = 0.78), *F*(1, 644) = 125.40, *p* < 0.001, *η^2^*_p_ = 0.16. Furthermore, participants in the vaccinated group (*M* = 1.45, *SD* = 0.86) reported higher levels of hate toward people with a different vaccination status than participants in the unvaccinated group (*M* = 0.55, *SD* = 0.48), *F*(1, 644) = 276.70, *p* < 0.001, $\eta_{p}^{2}$ = 0.30. These findings support Hypotheses 1a and 1b.

##### Cognitive polarization

For morality perceptions, we conducted a repeated measures ANOVA with vaccination status group as the between‐subjects factor and morality perceptions (vaccinated and unvaccinated) as the within‐subjects factor. The interaction effect was significant, *F*(1, 644) = 215.00, *p* < 0.001, $\eta_{p}^{2}$ = 0.25. The vaccinated group (*M* = 1.59, *SD* = 0.78) viewed unvaccinated people as significantly less moral compared to the unvaccinated group (*M* = 2.64, *SD* = 0.81), *F*(1, 644) = 280.10, *p* < 0.001, $\eta_{p}^{2}$ = 0.30. However, the vaccinated (*M* = 2.63, *SD* = 0.67) and unvaccinated groups (*M* = 2.55, *SD* = 0.76) did not differ in how moral they perceived vaccinated people, *F*(1, 644) = 1.84, *p* = 0.176, $\eta_{p}^{2}$ = 0.003.

For competence perceptions, the interaction effect was also significant, *F*(1, 644) = 237.00, *p* < 0.001, $\eta_{p}^{2}$ = 0.27. The unvaccinated group (*M* = 2.47, *SD* = 0.86) viewed vaccinated people as less competent compared to the vaccinated group (*M* = 2.60, *SD* = 0.66), *F*(1, 644) = 4.42, *p* = 0.036, $\eta_{p}^{2}$ = 0.007. However, the vaccinated group (*M* = 1.58, *SD* = 0.75) viewed unvaccinated people as less competent compared to the unvaccinated group (*M* = 2.68, *SD* = 0.84), *F*(1, 644) = 302.10, *p* < 0.001, $\eta_{p}^{2}$ = 0.32. These findings provide support for Hypothesis 1c for the effect of vaccination status on morality perceptions but not for competence perceptions. The vaccinated group viewed unvaccinated people as less competent, to a greater extent than vice versa.

**FIGURE 1:**
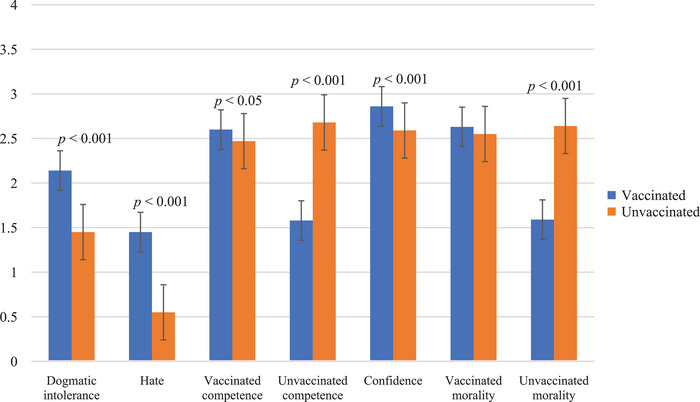
The bar graph compares the mean scores for six polarization indicators—dogmatic intolerance, hate, competence (for vaccinated and unvaccinated individuals), confidence, and morality (for vaccinated and unvaccinated individuals)—based on vaccination status. The blue bars represent responses from the vaccinated group, and the orange bars represent responses from the unvaccinated group. Error bars represent the standard error of the mean. The vaccinated group reported higher levels of dogmatic intolerance, hate, competence (of vaccinated individuals), confidence, and morality (of vaccinated individuals). Conversely, the unvaccinated group reported higher levels of competence (of unvaccinated individuals) and morality (of unvaccinated individuals). No significant difference is observed in the perceived morality of vaccinated people for either group.

We assessed confidence in knowledge by assessing confidence while controlling for factual COVID‐19 vaccine knowledge. Vaccinated people (*M* = 8.06, *SD* = 1.07) had higher factual knowledge than unvaccinated people (*M* = 6.13, *SD* = 1.94), *F*(1, 644) = 240.20, *p* < 0.001, $\eta_{p}^{2}$ = 0.27. Even after statistically controlling for factual knowledge, however, the vaccinated group (*M* = 2.86, *SD* = 0.57) reported higher confidence in their knowledge than the unvaccinated group (*M* = 2.59, *SD* = 0.57), *F*(1, 643) = 27.23, *p* < 0.001, $\eta_{p}^{2}$ = 0.04. The findings do not support Hypothesis 1d as the vaccinated group had both higher factual knowledge and higher confidence in that knowledge than the unvaccinated group. Figure 1 displays an overview of the effects of vaccination status on the polarization indicators.

Additionally, after applying Holm–Bonferroni correction for multiple comparisons, all previously significant (*p* < 0.05) results remained significant, and nonsignificant comparisons remained unchanged.

#### Conspiracy beliefs and polarization

Overall, the unvaccinated group had stronger conspiracy beliefs (*M* = 2.71, *SD* = 1.16) than the vaccinated group (*M* = 0.91, *SD* = 0.95), *F*(1, 644) = 481.10, *p* < 0.001, $\eta_{p}^{2}$ = 0.43. We therefore mean‐centered conspiracy beliefs within the vaccinated and unvaccinated groups separately to make vaccination status and conspiracy beliefs independent as predictors in the regression analyses, thus avoiding multicollinearity problems.

##### Affective polarization

Vaccination status had a significant main effect on dogmatic intolerance, *B* = 0.34, *SE* = 0.03, *t*(642) = 11.73, *p* < 0.001, CI_95%_ [0.29, 0.40]. Conspiracy beliefs did not have a significant effect on dogmatic intolerance, *B* = −0.03, *SE* = 0.03, *t*(642) = −0.98, *p* = 0.328, CI_95%_ [−0.08, 0.02]. Moreover, both vaccination status, *B* = 0.45, *SE* = 0.03, *t*(642) = 17.37, *p* < 0.001, CI_95%_ [0.40, 0.50], and conspiracy beliefs, *B* = −0.06, *SE* = 0.03, *t*(642) = −2.26, *p* = 0.024, CI_95%_ [−0.11, −0.01], had a significant main effect on hate.

More importantly, however, was that we found significant interaction effects of conspiracy beliefs and vaccination status on dogmatic intolerance, *B* = −0.23, *SE* = 0.03, *t*(642) = −8.01, *p* < 0.001, CI_95%_ [−0.29, −017], and hate, *B* = −0.20, *SE* = 0.03, *t*(642) = −7.75, *p* < 0.001, CI_95%_ [−0.25, −0.15]. Simple slope analyses revealed that higher conspiracy beliefs were associated with higher dogmatic intolerance*, B* = 0.20, *SE* = 0.04, *t*(335) = 5.54, *p* < 0.001, CI_95%_ [0.13, 0.27], and hate in the unvaccinated group, *B* = 0.14, *SE* = 0.02, *t*(335) = 6.34, *p* < 0.001, CI_95%_ [0.10, 0.18]. Conversely, lower conspiracy beliefs were associated with higher dogmatic intolerance, *B* = −0.26, *SE* = 0.04, *t*(307) = −5.84, *p* < 0.001, 95 CI_95%_ [−0.35, −0.17], and hate in the vaccinated group, *B* = −0.25, *SE* = 0.05, *t*(307) = −5.13, *p* < 0.001, 95% CI [−0.35, −0.16].

##### Cognitive polarization

Vaccination status had a significant main effect on morality perceptions of unvaccinated people, *B* = −0.53, *SE* = 0.03, *t*(642) = −17.44, *p* < 0.001, CI_95%_ [−0.58, −0.47], but not on morality perceptions of vaccinated people, *B* = 0.04, *SE* = 0.03, *t*(642) = 1.38, *p* = 0.169, CI_95%_ [−0.02, 0.09]. Conspiracy beliefs had a significant effect on morality perceptions of vaccinated people, *B* = −0.12, *SE* = 0.03, *t*(642) = −4.42, *p* < 0.001, CI_95%_ [−0.18, −0.07], and unvaccinated people, *B* = 0.22, *SE* = 0.03, *t*(642) = 7.45, *p* < 0.001, CI_95%_ [0.16, 0.28]. We did not find a significant interaction effect of conspiracy beliefs and vaccination status on how moral participants perceived vaccinated people, *B* = 0.02, *SE* = 0.03, *t*(642) = 0.62, *p* = 0.535 CI_95%_ [−0.04, 0.07], and unvaccinated people, *B* = 0.01, *SE* = 0.03, *t*(642) = 0.20, *p* = 0.840, CI_95%_ [−0.05, 0.06].

Vaccination status had a significant main effect on competence perceptions of vaccinated people, *B* = 0.06, *SE* = 0.03, *t*(642) = 2.15, *p* = 0.032, CI_95%_ [0.01, 0.12], and unvaccinated people, *B* = −0.55, *SE* = 0.03, *t*(642) = −18.48, *p* < 0.001, CI_95%_ [−0.61, −0.49]. Conspiracy beliefs had a significant effect on competence perceptions of vaccinated people, *B* = −0.14, *SE* = 0.03, *t*(642) = −4.80, *p* < 0.001, CI_95%_ [−0.20, −0.08], and unvaccinated people, *B* = 0.26, *SE* = 0.03, *t*(642) = 8.91, *p* < 0.001, CI_95%_ [0.20, 0.32]. There was also no significant interaction effect on how competent participants perceived vaccinated people, *B* = 0.05, *SE* = 0.03, *t*(642) = 1.59, *p* = 0.112, CI_95%_ [−0.01, 0.10], and unvaccinated people, *B* = −0.02, *SE* = 0.03, *t*(642) = −0.57, *p* = 0.569, CI_95%_ [−0.07, 0.04].

Both vaccination status, *B* = 0.20, *SE* = 0.03, *t*(641) = 6.39, *p* < 0.001, CI_95%_ [0.14, 0.27], and conspiracy beliefs, *B* = −0.08, *SE* = 0.03, *t*(641) = −2.84, *p* < 0.01, CI_95%_ [−0.14, −0.03], had a significant main effect on confidence in knowledge while controlling for factual knowledge. There was a significant interaction effect of conspiracy beliefs and vaccination status on confidence in knowledge while controlling for factual knowledge, *B* = −0.11, *SE* = 0.03, *t*(641) = −4.20, *p* < 0.001, CI_95%_ [−0.16, −0.06]. Simple slope analyses revealed that higher conspiracy beliefs were associated with higher confidence in knowledge in the unvaccinated group, *B* = 0.10, *SE* = 0.03, *t*(335) = 3.00, *p* = 0.003, CI_95%_ [0.04, 0.17]. Conversely, lower conspiracy beliefs were associated with higher confidence in knowledge in the vaccinated group, *B* = −0.16, *SE* = 0.03, *t*(307) = −4.77, *p* < 0.001, CI_95%_ [−0.22, −0.09].

Altogether, these findings support Hypotheses 2 and 3 for the interaction effect of conspiracy beliefs and vaccination status on dogmatic intolerance, hate, and confidence in knowledge, but not for their effects on morality and competence perceptions.

### Study 1: Discussion

Overall, the results of Study 1 suggest that in general, vaccinated people appear more polarized toward unvaccinated people than vice versa as reflected in their higher levels of affective and cognitive polarization toward unvaccinated people. Conspiracy beliefs moderated such polarization across vaccination status groups with the exception of morality and competence perceptions. Higher conspiracy beliefs among unvaccinated people predicted increased intolerance toward people with differing views about COVID‐19 vaccines and hate toward vaccinated others, and overconfidence in COVID‐19 vaccine knowledge. Among vaccinated people, lower conspiracy beliefs predicted increased intolerance toward people with differing views about COVID‐19 vaccines and hate toward unvaccinated people, and higher confidence in knowledge. We further found that vaccinated people expressed more liberal political views compared to unvaccinated people, who were more conservative.

### Study 2: Materials and methods

Study 2 sought to examine the possibility that unvaccinated people may have been polarized as well, but in a different manner than vaccinated people. Prior research has shown that unvaccinated people viewed the COVID‐19 vaccine as threatening,^29^, ^30^, ^31^ whereas vaccinated people viewed unvaccinated others as threatening.^8^, ^26^ Of importance, research has suggested that due to policies that pressured people to get vaccinated (e.g., excluding unvaccinated people from public places), unvaccinated people felt coerced by the government, which in turn fueled their conspiracy beliefs about power holders and their polarization.^6^ We therefore expected that unvaccinated people mostly were polarized toward the government, who were issuing the vaccine, rather than toward people who chose to get vaccinated. We tested this idea on the polarization indicator that arguably reflects the most extreme levels of outgroup animosity, namely, feelings of hate.^24^ We predicted that while vaccinated people would show stronger hatred toward unvaccinated people than vice versa (Hypothesis 1), unvaccinated people would show stronger hatred toward the government than vaccinated people would (Hypothesis 2). Exploratively, we sought to explore whether vaccinated people favored policies to exclude unvaccinated people from public places due to public health concerns, feelings of hate for the unvaccinated, or both.

#### Sample

Recruitment took place via Prolific in August 2022. The sample consisted of 503 participants from the United States (48.91% men, 48.91% women, 1.98% nonbinary/third gender) between the ages of 18 and 83 years old (*M*_age_ = 38.76, *SD*_age_ = 13.20). According to a sensitivity power analysis for the effect of vaccination status on hate (Hypothesis 1), this sample size allowed us to detect *f* = 0.18 with 80% power at 0.05 (*α* = 0.05). We sampled 259 vaccinated and 244 unvaccinated participants. Participants were recruited based on their vaccination status using the Prolific prescreening function. The study lasted about 5 min and participants were paid 0.70 UKP £ (approximately 0.90 USD). All participants gave their informed consent.

#### Procedure

We first assessed hate toward a vaccination status outgroup (vaccinated or unvaccinated) using the same 12‐item scale from Study 1.^52^ Additionally, participants completed a further scale assessing feelings of hate toward the government, example item: “I could not control my anger toward the government” (*α*_hate toward vaccinated people_ = 0.84, *α*_hate toward unvaccinated people_ = 0.93, *α*_hate toward government_ = 0.92). Public health concern was assessed on a 4‐item scale, example item: “How worried are you about the elderly and the immune‐compromised in the COVID‐19 pandemic?” (0 = *extremely unworried*, 4 = *extremely worried*; *α* = 0.91). We then assessed support for policies that exclude unvaccinated people using 6 items, example item: “Making proof of vaccination mandatory to board an airplane” (*α* = 0.98). Participants rated their agreement of these policies on a 5‐point scale (0 = *strongly disagree*, 4 = *strongly agree*). For completed scale items, see the Supporting Information. Lastly, participants provided basic demographics.

### Study 2: Results

While the primary analyses reported below were without covariates (which was in line with our preregistration), an ANOVA found that participants in the vaccinated group (*M* = 2.44, *SD* = 2.26) reported more liberal views, whereas participants in the unvaccinated group (*M* = 4.97, *SD* = 2.29) reported more conservative views, *F*(1, 501) = 156.00, *p* < 0.001, $\eta_{p}^{2}$ = 0.24. We therefore ran the primary analyses with political orientation, gender, and age as covariates to test the robustness of our results and reduce omitted‐variable bias. These results can be found in the Supporting Information. All hypotheses were still supported after including these covariates.

#### Effect of vaccination status on hate

The vaccinated group (*M* = 1.67, *SD* = 0.95) reported significantly higher levels of hate toward people with a different vaccination status than the unvaccinated group (*M* = 0.60, *SD* = 0.47), *F*(1, 501) = 247.20, *p* < 0.001, $\eta_{p}^{2}$ = 0.33. The unvaccinated group (*M* = 1.93, *SD* = 0.97) reported significantly higher levels of hate toward the government than the vaccinated group (*M* = 1.68, *SD* = 0.96), *F*(1, 501) = 8.21, *p* = 0.004, $\eta_{p}^{2}$ = 0.02. These findings support Hypotheses 1 and 2.

#### Support for excluding unvaccinated people

In a more exploratory fashion, we then conducted a linear regression analysis among vaccinated people to test predictors of their support for excluding unvaccinated people from public places.^c^ Results indicated that not only public health concern, *B* = 0.55, *SE* = 0.08, *t*(256) = 6.97, *p* < 0.001, CI_95%_ [0.40, 0.71], but also feelings of hate toward the unvaccinated, *B* = 0.60, *SE* = 0.06, *t*(256) = 9.37, *p* < 0.001, CI_95%_ [0.47, 0.72], were associated with increased support for excluding unvaccinated people among the vaccinated group.

### Study 2: Discussion

Overall, the results of Study 2 suggest that while vaccinated people demonstrate higher hate toward unvaccinated people than vice versa, unvaccinated people demonstrate higher hate toward the government. Furthermore, vaccinated people support excluding unvaccinated people from public places not only due to public health concern but also due to their feelings of hate toward them. As in Study 1, vaccinated people reported more liberal views, whereas unvaccinated people reported more conservative views.

## GENERAL DISCUSSION

The present study found that polarization manifests differently in vaccinated versus unvaccinated people. In line with previous findings,^7^, ^8^, ^26^ the results indicated that vaccinated people were polarized more toward unvaccinated others across both affective and cognitive dimensions. In Study 1 we expected vaccinated people to demonstrate greater dogmatic intolerance and hate, and lower morality perceptions toward unvaccinated people than vice versa. However, vaccinated people also showed overconfidence in their vaccine knowledge and lower competence perceptions toward unvaccinated people than vice versa. These findings suggest that across affective and cognitive dimensions, vaccinated people polarize more strongly toward unvaccinated people than vice versa. In a second study, we found evidence that unvaccinated people were affectively polarized as well, but toward a different target group. They expressed more hatred toward the government than vaccinated people did. This is in line with earlier findings suggesting that polarization among vaccinated people was associated with perceived coercion to take the vaccine.^6^ In an exploratory analysis among vaccinated people, we found that not only public health concerns but also feelings of hate predicted increased support for excluding unvaccinated people from public places. Altogether these findings suggest that both vaccinated and unvaccinated people were polarized, but toward different targets.

Study 1 also examined the moderating role of conspiracy theories in the polarization between vaccinated and unvaccinated people. Among unvaccinated people, conspiracy beliefs predicted higher dogmatic intolerance and hate toward vaccinated people, and increased confidence in COVID‐19 vaccine knowledge. Among vaccinated people, however, lower conspiracy beliefs predicted higher dogmatic intolerance and hate toward unvaccinated people, and increased confidence in COVID‐19 vaccine knowledge. Apparently, rejecting conspiracy theories is associated with rejecting unvaccinated people, which is consistent with findings suggesting that many citizens tend to reject others for expressing irrational beliefs or behaviors.^5^, ^6^, ^50^, ^51^ Conspiracy beliefs did not affect morality and competence perceptions for either vaccinated or unvaccinated people. These findings suggest that conspiracy beliefs were an important factor to understand why vaccinated and unvaccinated people were polarized.

The findings have a number of theoretical and practical implications. First, they underscore that in societal crisis situations, polarization may not be a dualistic process between two groups. During the COVID‐19 pandemic vaccinated people, unvaccinated people, and the government played a role in the polarization of society. While vaccinated people were polarized mostly toward the unvaccinated, across countries unvaccinated people protested against the restrictive measures. This is in line with our finding that unvaccinated people were mostly polarized toward the government, as well as with other findings that unvaccinated people felt coerced.^6^ This finding can be used to inform policy development when it comes to societal crisis situations that may yield excessive polarization between societal subgroups. For example, the decision to exclude unvaccinated people from public spaces can be seen as a trade‐off between protecting public health versus allowing for disruptive societal effects in the form of increased polarization. When faced with such difficult trade‐offs, campaigns that humanize both groups and foster mutual understanding may be especially important. Emphasizing shared values (e.g., safety, freedom) should be emphasized rather than reinforcing divisions. In particular, acknowledging concerns as valid and avoiding moral framing can mitigate divisiveness.^54^

Another important factor to consider is the risk of a breakdown in trust in public health systems, which may have implications that range far beyond the pandemic. To mitigate this distrust, it pays to invest in transparency in policymaking and engagement in two‐way communication in the future.

Second, the findings underscore the polarizing qualities of conspiracy theories. Whereas previous research has mostly focused on how polarization is associated with increased conspiracy beliefs,^44^, ^48^, ^55^ the present findings underscore that rejecting conspiracy theorists for their irrational beliefs may also contribute to polarization.^50^, ^51^ This finding underscores the importance of combating misinformation, especially in crisis situations. The findings point to a critical need for interventions to combat conspiracy beliefs and improve public understanding of vaccines. Media literacy initiatives can be effective, but timing is important. Pre‐emptive warnings about misleading information built resistance to conspiratorial thinking when exposure occurred.^56^

### Strengths and limitations

This study has various strengths and limitations. Among the strengths are that both studies were preregistered and well‐powered, suggesting that the results are reliable. Also, the present studies investigated intergroup polarization in the context of a real‐life health crisis and has clear relevance for understanding current societal events.

There are also several limitations that need to be noted, however. One limitation is that the data were collected in only one country, namely, the United States. It is necessary to examine how polarization manifests across different cultural contexts, such as more collectivistic cultures. One study found largely comparable dynamics in the polarization between vaccinated versus unvaccinated people in the United States, the United Kingdom, and China.^6^ Nevertheless, more research is needed to establish the cross‐cultural robustness of these effects.

A second limitation is that the research was conducted at a time when vaccination status still was a highly salient group identity. In the meantime, however, most countries (including the United States) have relaxed their pandemic policies and no longer require proof of vaccination when entering public spaces. As such, the current findings may no longer reflect the current attitudes of people who did, versus did not, decide to get vaccinated during the COVID‐19 pandemic. An interesting avenue for future research could be to investigate how time has affected mutual perceptions between vaccinated and unvaccinated people as the world has moved into a postpandemic era. This would be relevant to establish the long‐term effects of intergroup polarization in the aftermath of societal crisis events.

## CONCLUSION

The COVID‐19 pandemic has forged a new social identity through vaccination status, as what was once a private identity became public. The current research explored what the polarization between these two opposing societal groups looked like. The results suggest that vaccinated people are more polarized toward unvaccinated people than vice versa, whereas unvaccinated people are more polarized toward the government than vaccinated people. Conspiracy theories appeared to have contributed in significant ways to the polarization of vaccinated and unvaccinated people. Altogether, these findings suggest that both vaccinated and unvaccinated people were polarized during the pandemic, although toward different targets and via different processes.

## AUTHOR CONTRIBUTIONS

Lisa Willemsen: Conceptualization, Formal analysis, Data curation, Investigation, Methodology, Project administration, Software, Validation, Visualization, Writing—original draft, Writing—review & editing. Chiara Serafini, Sümeyye Ergün, John Butler: Conceptualization, Formal analysis, Data curation, Investigation, Methodology, Project administration, Visualization. Jan‐Willem van Prooijen: Conceptualization, Funding acquisition, Investigation, Methodology, Project administration, Resources, Supervision, Writing—review & editing

## CONFLICT OF INTEREST STATEMENT

The authors declare no conflicts of interest.

## Peer Review

The peer review history for this article is available at https://publons.com/publon/10.1111/nyas.15408

## Supporting information



Supporting information

## Footnotes

b We also had a preregistered hypothesis stipulating that public health concern would moderate the effects of polarization among vaccinated people, for which we found some support. To streamline and focus the current paper, we report the measurement and statistical results in the Supporting Information.

c See the Supporting Information for the correlation matrix.
