# Supplementary material for: Polarization in the COVID‐19 pandemic: The impact of vaccination status and conspiracy theories
Source: Ann N Y Acad Sci. 2025 Aug 4;1551(1):270–9. doi: 10.1111/nyas.15408 (PMC12448270; doi:10.1111/nyas.15408)
Supplement: Supplementary file 1 — Supporting information [file NYAS-1551-270-s001.docx]

**Supporting information**

| ***Contents overview***   1. Study 1: Survey scale items (pg. 2-7) 2. Study 2: Survey scale items (pg. 8-11) 3. Table S1: Quiz item-level difficulty for Study 1 (pg. 12) 4. Table S2: Demographics for Study 1 (pg. 13) 5. Study 1: Main analyses with covariates (pg. 14-17) 6. Study 2: Main analyses with covariates (pg. 18) 7. Study 1: Measurement invariance analysis for dogmatic intolerance scale (pg. 19) 8. Study 1: Public health concern and polarization (pg. 20-21) 9. Study 2: Exploratory analysis correlation matrix (pg. 22) |
| --- |

**Study 1: Survey scale items**

| ***COVID-19 Knowledge*** | |
| --- | --- |
| Question: How knowledgeable are you about the COVID-19 vaccines? | [4-Extremely knowledgeable]  [3-Very knowledgeable]  [2-Moderately knowledgeable]  [1-Slightly knowledgeable]  [0-Not knowledgeable at all] |
| Instruction: Please answer the following statements with true or false:   1. Getting vaccinated prevents you from contracting COVID-19. 2. Vaccinations must be approved by the World Health Organization (WHO). 3. Potential side effects of the COVID-19 vaccines are still being investigated by national and international bodies. 4. Scientific studies and tests about COVID-19 vaccine efficacy are accessible only from authorized people. 5. COVID-19 vaccines alter the DNA of the recipient. 6. Due to the vaccines, you can test positive for COVID-19 for a few days after receiving it. 7. Higher vaccination rates lower the odds of COVID-19 mutating further. 8. The vaccines increase the body’s ability to recognize the COVID-19 virus and develop a natural defense against it. 9. Due to the COVID-19 vaccines being developed so rapidly, they have not undergone rigorous, multi-stage testing. 10. People who have been vaccinated are less likely to get severely ill or die from COVID-19. | [1-True]  [0-False] |
| After each knowledge statement (see above) was presented, participants were asked:  How confident are you about your answer? | [4-Extremely confident]  [3-Very confident]  [2-Moderately confident]  [1-Slightly confident]  [0-Not confident at all] |

| ***Dogmatic Intolerance (edited from van Prooijen & Krouwel, 2017)*** | |
| --- | --- |
| Instruction: Please rate your level of agreement with the following statements.   1. I believe everyone should think like me about the COVID-19 vaccines. 2. If everyone would think about it, they would hold the same opinions I do about the COVID-19 vaccines. 3. How I feel about the COVID-19 vaccines is the truth. 4. People who think differently than me when it comes to the COVID-19 vaccines are of lesser value than I am. 5. It scares me if people think differently than I do about the COVID-19 vaccines. 6. I never really encounter people who think differently than I do about the COVID-19 vaccines. | [4-Strongly agree]  [3-Somewhat agree]  [2-Neither agree nor disagree]  [1-Somewhat disagree]  [0-Strongly disagree] |

| ***Hate for Unvaccinated People (edited from Zeki & Romaya, 2008)*** | |
| --- | --- |
| Instruction: Please think about the group of people who have decided not to get vaccinated, and indicate the extent to which you agree or disagree with each of the following statements.   1. I cannot control my anger towards unvaccinated people. 2. I would like to do something to harm unvaccinated people. 3. I have aggressive thoughts about unvaccinated people. 4. I have kind thoughts about unvaccinated people (Recoded). 5. Unvaccinated people are scum. 6. I really despise unvaccinated people. 7. Unvaccinated people do not deserve any consideration. 8. Unvaccinated people are very nice (Recoded). 9. I don’t want any unvaccinated people near me. 10. The world would be a better place without unvaccinated people. 11. Unvaccinated people are really disgusting. 12. I would like to interact with unvaccinated people (Recoded). | [4-Strongly agree]  [3-Somewhat agree]  [2-Neither agree nor disagree]  [1-Somewhat disagree]  [0-Strongly disagree] |
| ***Hate for Vaccinated People (edited from Zeki & Romaya, 2008)*** | |
| Instruction: Please think about the group of people who have decided to get vaccinated, and indicate the extent to which you agree or disagree with each of the following statements.   1. I cannot control my anger towards vaccinated people. 2. I would like to do something to harm vaccinated people. 3. I have aggressive thoughts about vaccinated people. 4. I have kind thoughts about vaccinated people (Recoded). 5. Vaccinated people are scum. 6. I really despise vaccinated people. 7. Vaccinated people do not deserve any consideration. 8. Vaccinated people are very nice (Recoded). 9. I don’t want any vaccinated people near me. 10. The world would be a better place without vaccinated people. 11. Vaccinated people are really disgusting. 12. I would like to interact with vaccinated people (Recoded). | [4-Strongly agree]  [3-Somewhat agree]  [2-Neither agree nor disagree]  [1-Somewhat disagree]  [0-Strongly disagree] |
| *Note.* The scale participants received was dependent on their vaccination status. Participants in the unvaccinated group received the Hate for Vaccinated People scale and participants in the vaccinated people received the Hate for Unvaccinated People scale. | |

| ***Morality and Competence Perceptions of Unvaccinated People (edited from Fiske et al., 2002)*** | |
| --- | --- |
| Instruction: Please rate your answers to the questions below.   1. How competent are people who choose to not get vaccinated? 2. How capable are people who choose to not get vaccinated? 3. How efficient are people who choose not to get vaccinated? 4. How intelligent are people who choose to not get vaccinated? 5. How skillful are people who choose to not get vaccinated? 6. How friendly are people who choose to not get vaccinated? 7. How well-intentioned are people who choose to not get vaccinated? 8. How trustworthy are people who choose to not get vaccinated? 9. How warm are people who choose to not get vaccinated? 10. How good-natured are people who choose to not get vaccinated? 11. How sincere are people who choose to not get vaccinated? | [4-Extremely high]  [3-Somewhat high]  [2- Neither low nor high]  [1-Somewhat low]  [0-Extremely low] |
| ***Morality and Competence Perceptions of Vaccinated People (edited from Fiske et al., 2002)*** | |
| Instruction: Please rate your answers to the questions below.   1. How competent are people who choose to get vaccinated? 2. How capable are people who choose to get vaccinated? 3. How efficient are people who choose to get vaccinated? 4. How intelligent are people who choose to get vaccinated? 5. How skillful are people who choose to get vaccinated? 6. How friendly are people who choose to get vaccinated? 7. How well-intentioned are people who choose to get vaccinated? 8. How trustworthy are people who choose to get vaccinated? 9. How warm are people who choose to get vaccinated? 10. How good-natured are people who choose to get vaccinated? 11. How sincere are people who choose to get vaccinated? | [4-Extremely high]  [3-Somewhat high]  [2- Neither low nor high]  [1-Somewhat low]  [0-Extremely low] |
| *Note.* Participants completed both scales regardless of whether they reported being vaccinated or not.   \| ***Conspiracy Beliefs*** \| \| \| --- \| --- \| \| Instruction: Please indicate your agreement with the following statements.   1. Pharmaceutical companies are dishonest about the possible dangers of the COVID-19 vaccines. 2. The COVID-19 vaccination campaign is being used by powerful people to serve their own interests. 3. The government purposefully exaggerates the effectiveness of the COVID-19 vaccines and underestimates the chance of harmful side-effects. 4. The COVID-19 vaccines are currently still in a far more experimental stage than is being told to citizens. 5. The COVID-19 vaccination campaign has little to do with protecting public health and is mostly about making money. 6. The government and pharmaceutical companies are dishonest about what is inside COVID-19 vaccines. \| [4-Strongly agree]  [3-Somewhat agree]  [2-Neither agree nor disagree]  [1-Somewhat disagree]  [0-Strongly disagree] \| | |

| ***Public Health Concern*** | |
| --- | --- |
| Instruction: Please answer the following questions.   1. How worried are you about the elderly and the immune-compromised in the COVID-19 pandemic? 2. How concerned are you about public health during the COVID-19 pandemic? 3. How concerned are you about your personal health during the COVID-19 pandemic? 4. How worried are you about how the COVID-19 pandemic is harming the health of your fellow citizens? | [4-Extremely worried]  [3-Somewhat worried]  [2-Neither unworried nor worried]  [1-Somewhat unworried]  [0-Extremely unworried] |

**Study 2: Survey scale items**

| ***Hate for Unvaccinated People (edited from Zeki & Romaya, 2008)*** | |
| --- | --- |
| Instruction: Please think about the group of people who decided not to get vaccinated during the COVID-19 pandemic, and remember how you felt towards this group throughout the course of the pandemic. Please indicate the extent to which you agree or disagree with each of the following statements.   1. I cannot control my anger towards unvaccinated people. 2. I would like to do something to harm unvaccinated people. 3. I have aggressive thoughts about unvaccinated people. 4. I have kind thoughts about unvaccinated people (Recoded). 5. Unvaccinated people are scum. 6. I really despise unvaccinated people. 7. Unvaccinated people do not deserve any consideration. 8. Unvaccinated people are very nice (Recoded). 9. I don’t want any unvaccinated people near me. 10. The world would be a better place without unvaccinated people. 11. Unvaccinated people are really disgusting. 12. I would like to interact with unvaccinated people (Recoded). | [4-Strongly agree]  [3-Somewhat agree]  [2-Neither agree nor disagree]  [1-Somewhat disagree]  [0-Strongly disagree] |
| ***Hate for Vaccinated People (edited from Zeki & Romaya, 2008)*** | |
| Instruction: Please think about the group of people who decided to get vaccinated during the COVID-19 pandemic, and remember how you felt towards this group throughout the course of the pandemic. Please indicate the extent to which you agree or disagree with each of the following statements.   1. I cannot control my anger towards vaccinated people. 2. I would like to do something to harm vaccinated people. 3. I have aggressive thoughts about vaccinated people. 4. I have kind thoughts about vaccinated people (Recoded). 5. Vaccinated people are scum. 6. I really despise vaccinated people. 7. Vaccinated people do not deserve any consideration. 8. Vaccinated people are very nice (Recoded). 9. I don’t want any vaccinated people near me. 10. The world would be a better place without vaccinated people. 11. Vaccinated people are really disgusting. 12. I would like to interact with vaccinated people (Recoded). | [4-Strongly agree]  [3-Somewhat agree]  [2-Neither agree nor disagree]  [1-Somewhat disagree]  [0-Strongly disagree] |
| ***Hate for Government (edited from Zeki & Romaya, 2008)*** | |
| Instruction: Please think about how the government reacted to the COVID-19 pandemic, and remember how you felt towards the government throughout the course of the pandemic. Please indicate the extent to which you agree or disagree with each of the following statements.   1. I cannot control my anger towards the government. 2. I would like to do something to harm the government 3. I have aggressive thoughts about the government. 4. I have kind thoughts about the government (Recoded). 5. The government are scum. 6. I really despise the government. 7. The government do not deserve any consideration. 8. The government are very nice (Recoded). 9. I don’t want any government representative near me. 10. The world would be a better place without the government. 11. The government are really disgusting. 12. I would like to interact with government representatives (Recoded). | [4-Strongly agree]  [3-Somewhat agree]  [2-Neither agree nor disagree]  [1-Somewhat disagree]  [0-Strongly disagree] |
| *Note.* Participants in the unvaccinated group received the Hate for Vaccinated People scale and participants in the vaccinated people received the Hate for Unvaccinated People scale. All participants received the Hate for Government scale. | |

| ***Public Health Concern*** | |
| --- | --- |
| Instruction: Please answer the following questions regarding health concerns during the COVID-19 pandemic.   1. How worried are you about the elderly and the immune-compromised in the COVID-19 pandemic? 2. How concerned are you about public health during the COVID-19 pandemic? 3. How concerned are you about your personal health during the COVID-19 pandemic? 4. How worried are you about how the COVID-19 pandemic is harming the health of your fellow citizens? | [4-Extremely worried]  [3-Somewhat worried]  [2-Neither unworried nor worried]  [1-Somewhat unworried]  [0-Extremely unworried] |

| ***Support for Excluding Unvaccinated People*** | |
| --- | --- |
| Instruction: In many U.S. states, and in many other countries worldwide, restrictions were imposed on unvaccinated people at some point during the pandemic. Here, we would like to ask how you have felt about some of the restrictions that have been imposed when there were many infections (in the U.S. or elsewhere).   1. Making proof of vaccination mandatory to board an airplane. 2. Making proof of vaccination mandatory to enter government buildings. 3. Making proof of vaccination mandatory to enter bars and restaurants. 4. Making proof of vaccination mandatory to enter non-essential stores. 5. Making proof of vaccination mandatory to enter indoor public places (i.e., museums, libraries). 6. Making proof of vaccination mandatory when crossing international borders. | [4-Strongly agree]  [3-Somewhat agree]  [2-Neither agree nor disagree]  [1-Somewhat disagree]  [0-Strongly disagree] |

| **Table S1**  *Quiz item-level difficulty for Study 1* | |
| --- | --- |
| Question (True/False) | Item-level difficulty |
| (1a) Getting vaccinated prevents you from contracting COVID-19. | 0.82 |
| (2a) Vaccinations must be approved by the World Health Organization (WHO). | 0.39 |
| (3a) Potential side effects of the COVID-19 vaccines are still being investigated by national and international bodies. | 0.96 |
| (4a) Scientific studies and tests about COVID-19 vaccine efficacy are accessible only from authorized people. | 0.71 |
| (5a) COVID-19 vaccines alter the DNA of the recipient. | 0.86 |
| (6a) Due to the vaccines, you can test positive for COVID-19 for a few days after receiving it. | 0.50 |
| (7a) Higher vaccination rates lower the odds of COVID-19 mutating further. | 0.64 |
| (8a) The vaccines increase the body’s ability to recognize the COVID-19 virus and develop a natural defense against it. | 0.83 |
| (9a) Due to the COVID-19 vaccines being developed so rapidly, they have not undergone rigorous, multi-stage testing. | 0.53 |
| (10a) People who have been vaccinated are less likely to get severely ill or die from COVID-19. | 0.83 |
| *Note.* This table gives the proportion of participants who answered the question correctly. | |

| **Table S2**  *Demographics for Study 1* | | |
| --- | --- | --- |
| Levels | Counts | % of total |
| Education | | |
| Primary education | 3 | 0.46 |
| Secondary education | 249 | 38.54 |
| Tertiary education | 300 | 46.44 |
| Postgraduate education | 94 | 14.55 |
| Political orientation | | |
| Liberal | 283 | 43.81 |
| Moderate | 164 | 25.39 |
| Conservative | 199 | 30.8 |

**Study 1: Main analyses with political orientation, gender, and age as covariates**

***Effect of vaccination status on polarization***

**Affective polarization.** Participants in the vaccinated group (*M* = 2.14, *SD* = 0.78) were more dogmatically intolerant about people with a different vaccination status than participants in the unvaccinated group (*M* = 1.45, *SD* = 0.78), *F*(1, 641) = 128.22, *p* < 0.001, *η^2^_p_* = 0.17. Furthermore, participants in the vaccinated group (*M* = 1.45, *SD* = 0.86) reported higher levels of hate towards people with a different vaccination status than participants in the unvaccinated group (*M* = 0.55, *SD* = 0.48), *F*(1, 641) = 285.35, *p* < 0.001, η^2^_p_ = 0.31. These findings support Hypothesis 1a and 1b.

**Cognitive polarization.** For morality perceptions, we conducted a repeated measures ANOVA with vaccination status group as the between-subjects factor and morality perceptions (vaccinated and unvaccinated) as the within-subjects factor. The interaction effect was significant, *F*(1, 641) = 104.13, *p* < 0.001, η^2^_p_ = 0.14. The vaccinated group (*M* = 1.59, *SD* = 0.78) viewed unvaccinated people as significantly less moral compared to the unvaccinated group (*M* = 2.64, *SD* = 0.81), *F*(1, 644) = 321.69, *p* < 0.001, η^2^_p_ = 0.33. However, the vaccinated (*M* = 2.63, *SD* = 0.67) and unvaccinated groups (*M* = 2.55, *SD* = 0.76) did not differ in how moral they perceived vaccinated people, *F*(1, 641) = 1.86, *p* = 0.176, η^2^_p_ = 0.003.

For competence perceptions, the interaction effect was also significant, *F*(1, 641) = 122.16, *p* < 0.001, η^2^_p_ = 0.16. The unvaccinated group (*M* = 2.47, *SD* = 0.86) viewed vaccinated people as less competent compared to the vaccinated group (*M* = 2.60, *SD* = 0.66), *F*(1, 641) = 4.49, *p* = 0.034, η^2^_p_ = 0.007. However, the vaccinated group (*M* = 1.58, *SD* = 0.75) viewed unvaccinated people as less competent compared to the unvaccinated group (*M* = 2.68, *SD* = 0.84), *F*(1, 644) = 345.70. *p* < 0.001, η^2^_p_ = 0.35. These findings provide support for Hypothesis 1c for the effect of vaccination status on morality perceptions but not for competence perceptions. The vaccinated group viewed unvaccinated people as less competent, to a greater extent than vice versa.

We assessed confidence in knowledge by assessing confidence while controlling for factual COVID-19 vaccine knowledge. Vaccinated people (*M* = 8.06, *SD* = 1.07) had higher factual knowledge than unvaccinated people (*M* = 6.13, *SD* = 1.94), *F*(1, 641) = 261.21, *p* < 0.001, η^2^_p_ = 0.29. Even after statistically controlling for factual knowledge, however, the vaccinated group (*M* = 2.86, *SD* = 0.57) reported higher confidence in their knowledge than the unvaccinated group (*M* = 2.59, *SD* = 0.57), *F*(1, 640) = 28.19, *p* < 0.001, η^2^_p_ = 0.04. The findings do not support Hypothesis 1d as the vaccinated group had both higher factual knowledge and higher confidence in that knowledge than the unvaccinated group. Figure 1 displays an overview of the effects of vaccination status on the polarization indicators.

***Conspiracy beliefs and polarization***

Overall, the unvaccinated group had stronger conspiracy beliefs (*M* = 2.71, *SD* = 1.16) than the vaccinated group (*M* = 0.91, *SD* = 0.95), *F*(1, 641) = 576.39, *p* < 0.001, η^2^_p_ = 0.47. We therefore mean-centered conspiracy beliefs within the vaccinated and unvaccinated groups separately to make vaccination status and conspiracy beliefs independent as predictors in the regression analyses, thus avoiding multicollinearity problems.

**Affective polarization.** Vaccination status had a significant main effect on dogmatic intolerance, *B* = 0.31, *SE* = 0.03, *t*(639) = 9.26, *p* < 0.001, CI_95%_ [0.24, 0.37]. Conspiracy beliefs did not have a significant effect on dogmatic intolerance, *B* = 0.01, *SE* = 0.03, *t*(639) = 0.44, *p* = 0.661, CI_95%_ [-0.05, 0.07]. Vaccination status had a significant main effect on hate, *B* = 0.41, *SE* = 0.03, *t*(639) = 14,14, *p* < 0.001, CI_95%_ [0.35, 0.47]. Conspiracy beliefs did not have a significant effect on hate, *B* = -0.02, *SE* = 0.03, *t*(639) = -0.66, *p* = 0.509, CI_95%_ [-0.07, 0.02].

More important, however, was that we found significant interaction effects of conspiracy beliefs and vaccination status on dogmatic intolerance, *B* = -0.25, *SE* = 0.03, *t*(639) = -8.74, *p* < 0.001, CI_95%_[-0.30, -019], and hate, *B* = -0.21, *SE* = 0.02, *t*(639) = -8.49, *p* < 0.001, CI_95%_ [-0.26, -0.16]. Simple slope analyses revealed that higher conspiracy beliefs predicted higher dogmatic intolerance*, B* = 0.23, *SE* = 0.04, *t*(332) = 5.49, *p* < 0.001, CI_95%_ [0.14, 0.31], and hate in the unvaccinated group, *B* = 0.14, *SE* = 0.02, *t*(332) = 5.75, *p* < 0.001, CI_95%_ [0.09, 0.19]. Conversely, lower conspiracy beliefs predicted higher dogmatic intolerance, *B* = -0.21, *SE* = 0.05, *t*(304) = -4.64, *p* < 0.001, 95 CI_95%_ [-0.30, -0.12], and hate in the vaccinated group, *B* = -0.18, *SE* = 0.05, *t*(304) = -3.41, *p* < 0.001, 95% CI [-0.28, -0.07].

**Cognitive polarization.** Vaccination status had a significant main effect on morality perceptions of unvaccinated people, *B* = -0.45, *SE* = 0.03, *t*(639) = -13.68, *p* < 0.001, CI_95%_ [-0.52, -0.39], but not on morality perceptions of vaccinated people*, B* = 0.02, *SE* = 0.03, *t*(639) = 0.75, *p* = 0.451, CI_95%_ [-0.04, 0.09]. Conspiracy beliefs had a significant effect on morality perceptions of vaccinated people, *B* = -0.12, *SE* = 0.03, *t*(639) = -3.95, *p* < 0.001, CI_95%_ [-0.18, -0.06], and unvaccinated people, *B* = 0.15, *SE* = 0.03, *t*(639) = 5.00, *p* < 0.001, CI_95%_ [0.09, 0.21]. We did not find a significant interaction effect of conspiracy beliefs and vaccination status on how moral participants perceived vaccinated people, *B* = 0.03, *SE* = 0.03, *t*(639) = 0.93, *p* = 0.351, CI_95%_[-0.03, 0.08], and unvaccinated people, *B* = 0.03, *SE* = 0.03, *t*(639) = 0.89, *p* = 0.372, CI_95%_[-0.03, 0.08].

Vaccination status had a significant main effect on competence perceptions of unvaccinated people, *B* = -0.49, *SE* = 0.03, *t*(639) = -14.84, *p* < 0.001, CI_95%_ [-0.55, -0.42], but not on competence perceptions of vaccinated people, *B* = 0.06, *SE* = 0.03, *t*(639) = 2.15, *p* = 0.088, CI_95%_ [-0.008, 0.13]. Conspiracy beliefs had significant effect on competence perceptions of vaccinated people, *B* = -0.15, *SE* = 0.03, *t*(639) = -4.60, *p* < 0.001, CI_95%_ [-0.21, -0.08], and unvaccinated people, *B* = 0.20, *SE* = 0.03, *t*(639) = 6.54, *p* < 0.001, CI_95%_ [0.14, 0.26]. There was also no significant interaction effect on how competent participants perceived vaccinated people, *B* = 0.06, *SE* = 0.03, *t*(639) = 1.91, *p* = 0.056, CI_95%_[-0.001, 0.11], and unvaccinated people, *B* = 0.004, *SE* = 0.03, *t*(639) = 0.14, *p* = 0.891, CI_95%_[-0.05, 0.06].

Both vaccination status, *B* = 0.20, *SE* = 0.03, *t*(638) = 5.81, *p* < 0.001, CI_95%_ [0.13, 0.26], and conspiracy beliefs, *B* = -0.08, *SE* = 0.03, *t*(638) = -2.48, *p* = 0.013, CI_95%_ [-0.14, -0.02], had a significant main effect on confidence in knowledge while controlling for factual knowledge. There was a significant interaction effect of conspiracy beliefs and vaccination status on confidence in knowledge while controlling for factual knowledge, *B* = -0.11, *SE* = 0.03, *t*(638) = -4.26, *p* < 0.001, CI_95%_[-0.16, -0.06]. Simple slope analyses revealed that higher conspiracy beliefs predicted higher confidence in knowledge in the unvaccinated group, *B* = 0.09, *SE* = 0.04, *t*(332) = 2.22, *p* = 0.003, CI_95%_[0.009, 0.16]. Conversely, lower conspiracy beliefs predicted higher confidence in knowledge in the vaccinated group, *B* = -0.16, *SE* = 0.03, *t*(304) = -4.47, *p* < 0.001, CI_95%_[-0.23, -0.09].

Altogether, these findings support Hypotheses 2 and 3 for the interaction effect of conspiracy beliefs and vaccination status on dogmatic intolerance, hate and confidence in knowledge, but not for their effects on morality and competence perceptions.

**Study 2: Main analyses with political orientation, gender and age as covariates**

***Effect of vaccination status on hate***

The vaccinated group (*M* = 1.67, *SD* = 0.95) reported significantly higher levels of hate towards people with a different vaccination status than the unvaccinated group (*M* = 0.60, *SD* = 0.47), *F*(1, 498) = 261.11, *p* < 0.001, η^2^_p_ = 0.34. The unvaccinated group (*M* = 1.93, *SD* = 0.97) reported significantly higher levels of hate towards the government than the vaccinated group (*M* = 1.68, *SD* = 0.96), *F*(1, 498) = 8.25, *p* = 0.004, η^2^_p_ = 0.02. These findings support Hypothesis 1 and 2.

***Support for excluding vaccinated people***

In a more exploratory fashion, we then conducted a linear regression analysis among vaccinated people to test predictors of their support for excluding unvaccinated people from public places, see Table S3. Results indicated that not only public health concern, *B* = 0.52, *SE* = 0.08, *t*(253) = 6.29, *p* < 0.001, CI_95%_[0.36, 0.68], but also feelings of hate towards the unvaccinated, *B* = 0.52, *SE* = 0.07, *t*(253) = 7.77, *p* < 0.001, CI_95%_[0.39, 0.65] predicted increased support for excluding unvaccinated people amongst the vaccinated group.

**Study 1: Measurement invariance analyses for dogmatic intolerance scale**

We conducted tests for configural invariance, metric invariance, and scalar invariance for the dogmatic intolerance scale across vaccination status groups. CFI (> 0.90), the RMSEA (< 0.08) and the SRMR (< 0.08) were considered indicators of acceptable model fit. The thresholds when testing for metric and scalar invariance were ΔCFI < -0.010; ΔRMSEA < 0.015; and ΔSRMR < 0.030. We tested an intolerance model including all six items of dogmatic intolerance. The basic configural model had an acceptable fit according to two out of three indicators (CFI = 0.946; RMSEA = 0.101, CI_90%_[0.08, 0.13]; SRMR = .046), *X^2^*^[18,^ *^N^* ^= 646]^ = 77.19, *p* < 0.001). The metric model did not deviate from the configural model according to all indicators (ΔCFI = -0.001; ΔRMSEA = -0.011; ΔSRMR = 0.008; Δ*X^2^*^[23,^ *^N^* ^= 646]^ = 83.68, *p* < 0.001). The scalar model did not deviate from the metric model according to two out of three indicators (ΔCFI = -0.019; ΔRMSEA = 0.005; ΔSRMR = 0.009), Δ*X^2^*^[28,^ *^N^* ^= 646]^ = 109.81, *p* < 0.001.

**Study 1: Public health concern and polarization**

We expected that among vaccinated people, higher public health concern would predict higher levels of affective polarization and morality perceptions. In Study 1, we measured public health concern on a 4-item scale, example item: ‘How worried are you about the elderly and the immune-compromised in the COVID-19 pandemic?’ (0 = *extremely unworried*, 4 = *extremely worried*; α = 0.91). Overall, the vaccinated group had stronger concern for public health (*M* = 3.20, *SD* = 0.75) than the unvaccinated group (*M* = 2.33, *SD* = 1.11), *F*(1, 644) = 132.00, *p* < 0.001, η^2^_p_ = 0.17. We therefore mean-centered public health concern within the vaccinated and unvaccinated groups separately to make the factors independent, avoiding multicollinearity problems.

***Affective polarization***

**Dogmatic intolerance.** Vaccination status had a significant main effect on dogmatic intolerance, *B* = 0.34, *SE* = 0.03, *t*(642) = 11.31, *p* < 0.001, CI_95%_ [0.28, 0.40], whereas public did not have a significant effect, *B* = 0.04, *SE* = 0.03, *t*(642) = 1.22, *p* = 0.225, CI_95%_ [-0.03, 0.11]. There was a significant interaction effect of public health concern and vaccination status on dogmatic intolerance, *B* = 0.13, *SE* = 0.03, *t*(642) = 3.85, *p* < 0.001, CI_95%_[0.07, 0.20]. Simple slope analyses revealed that public health concern positively predicted intolerance, *B* = 0.18, *SE* = 0.06, *t*(307) = 3.05, *p* = 0.003, CI_95%_[0.06, 0.29], in the vaccinated group and negatively predicted intolerance, *B* = -0.09, *SE* = 0.04, *t*(335) = -2.40, *p* = 0.017, CI_95%_[-0.17, -0.02], in the unvaccinated group.

**Hate.** Vaccination status had a significant main effect on hate, *B* = 0.45, *SE* = .03, *t*(642) = 16.92, *p* < 0.001, CI_95%_ [0.40, 0.50], whereas public health concern did not have a significant effect, *B* = 0.06, *SE* = 0.03, *t*(642) = 1.81, *p* = 0.070, CI_95%_ [-0.00, 0.11]. There was a significant interaction effect of public health concern and vaccination status on hate, *B* = 0.15, *SE* = 0.03, *t*(642) = 4.90, *p* < 0.001, CI_95%_[0.09, 0.21]. Simple slope analyses revealed that public health concern positively predicted hate, *B* = 0.20, *SE* = 0.06, *t*(307) = 3.20, *p* = 0.002, CI_95%_[0.08, 0.33], in the vaccinated group. Public health concern negatively predicted hate, *B* = -0.09, *SE* = 0.02, *t*(335) = -4.09, *p* < 0.001, CI_95%_[-0.14, -0.05], in the unvaccinated group.

***Morality perceptions***

Vaccination status had a significant main effect on morality perceptions of unvaccinated people, *B* = -0.53, *SE* = 0.03, *t*(642) = -16.90, *p* < 0.001, CI_95%_ [-0.59, -0.46], but not on morality perceptions of vaccinated people, *B* = 0.04, *SE* = 0.03, *t*(642) = 1.39, *p* = 0.165, CI_95%_ [-0.02, 0.09]. Public health concern had a significant effect on morality perceptions of vaccinated people, *B* = 0.19, *SE* = 0.03, *t*(642) = 5.87, *p* < 0.001, CI_95%_ [0.13, 0.25], and unvaccinated people, *B* = -0.13, *SE* = 0.03, *t*(642) = -16.90, *p* < 0.001, CI_95%_ [-0.20, -0.06]. The interaction effect of public health concern and vaccination status (vaccinated group) on morality perceptions of unvaccinated people was non-significant, *B* = -0.00, *SE* = 0.04, *t*(642) = -0.012, *p* = 0.990, CI_95%_[-0.07, 0.07]. Hypothesis 4 was supported for the effect of public health concern and vaccination status on dogmatic intolerance and hate but not for morality perceptions of unvaccinated people.

**Study 2: Exploratory analysis correlation matrix**

All three variables (support for excluding unvaccinated people, public health concern, and hate towards unvaccinated people) correlated positively with each other, see Table S2.

| **Table S3**  *Means, standard deviations and correlations for exploratory analysis* | | | | |
| --- | --- | --- | --- | --- |
| Variables | *M* | *SD* | 1 | 2 |
| 1. Support for excluding unvaccinated people | 2.90 | 1.22 |  |  |
| 2. Public health concern | 3.27 | 0.76 | 0.512*** |  |
| 3. Hate towards unvaccinated people | 1.67 | 0.95 | 0.588*** | 0.357*** |
| *Note.* * *p* < 0.05; ** *p* < 0.01; *** *p* < 0.001. | | | | |
